# Mandibular trabecular bone pattern before and two years after medical or surgical obesity treatment in young Swedish women

**DOI:** 10.1007/s00784-024-06142-y

**Published:** 2025-01-12

**Authors:** Anna-Lena Östberg, Ville Wallenius, Negin Taghat, Grethe Jonasson

**Affiliations:** 1 Department of Behavioral and Community Dentistry, Institute of Odontology, Sahlgrenska Academy, University of Gothenburg, P.O. Box 450, Gothenburg, SE-40530 Sweden; 2 Department of Surgery, Institute of Clinical Sciences, Sahlgrenska Academy, University of Gothenburg, Gothenburg, Sweden; 3 Department of Surgery, Sahlgrenska University Hospital, Region Västra Götaland, Gothenburg, Sweden

**Keywords:** Bariatrics, Bone density, Mandible, Radiography

## Abstract

**Objective:**

To investigate if changes in body mass index (BMI) result in changes of the mandibular trabecular bone structure.

**Materials and methods:**

Females (18–35 years at baseline, mean BMI 42,3) were followed from before (*n* = 117) until two years (*n* = 66) after obesity treatment (medical or surgical). The mandibular bone trabeculation was classified as sparse, dense, or mixed on intraoral radiographs (Lindh’s index). A digitized method (Jaw-X) assessed the size and intensities of intertrabecular spaces. The main predictor variable was BMI reduction over the period.

**Results:**

Before treatment, the group with a high BMI (≥ 45) had a significantly denser bone than those with a lower BMI (*p* = 0.035). Two years after treatment, fewer were classified with sparse bone (Lindh’s index *p* = 0.001, Jaw-X *p* = 0.009). The physical activity increased with fewer having a sedentary lifestyle (40% before, 17% after treatment). The association between BMI reduction and the difference in Jaw-X was significant in regression models and not influenced by obesity treatment method but by baseline factors as age, trabecular bone pattern and level of ionized calcium.

**Conclusions:**

Before obesity treatment, high BMI was associated with dense bone trabeculation in the jaw. The group with sparse bone had decreased at follow-up. The association between BMI reduction and bone trabeculation was influenced by individual and medical factors.

**Clinical relevance:**

Bone trabeculation in the mandible was maintained during the first years after obesity treatment but new health habits should be encouraged, and patients need to be monitored and followed up further.

**Supplementary Information:**

The online version contains supplementary material available at 10.1007/s00784-024-06142-y.

## Introduction

Obesity is an increasing health problem and in 2020, 14 per cent of adults worldwide were reported as obese with a Body Mass Index (BMI) exceeding 30 kg/m^2^ [1]. It is a complex disease where both lifestyle and medical factors have been shown to be risk factors for high BMI [2]. In turn, obesity is a risk factor for increased morbidity and mortality [3, 4]. Both medical and surgical treatment reduce these risks [5], with bariatric surgery being the most effective for weight loss [6, 7]. For oral health, associations between obesity and periodontal disease have been observed [8] and higher caries levels with increasing BMI have been shown [9].

However, there are side effects of bariatric surgery, both short-term post-operative complications and long-term complications such as malnutrition [10–12] and increased fracture risk [13–16]. Whether medical treatment for obesity affects bone health has not been investigated in the same way.

The mechanisms that determine bone strength are complex, including variables such as the shape and size of the bone, bone mass, collagen content and the microstructure, e.g., the lamellae [17,18]. Bone modelling dominates at young ages, whereas remodeling continues throughout life. During the bone remodeling process, old bone is replaced with new bone [17]. Throughout life, bone is continuously affected, e.g., by nutritional status, hormones, and the concentration of circulating calcium in the blood [19]. Mechanical load is another factor of interest as weight-bearing physical activity is needed to maintain bone density [20]. Weightlessness and inactivity result in decreased skeletal bone mass and density [21]. In one study, weightlifters had significantly higher bone mass in the hip and spine compared with controls, but lower bone mass in the skull [22]. Research into skeletal health has often focused on women before and after the menopause as the oestrogen level then decreases causing decreased bone mass and increased risk for osteoporosis [19]. The significance for bone mass of a high body weight, as in obesity, is less well understood.

The factors that affect bone health are thus many and complex, which makes it difficult to estimate bone strength. However, previous research has indicated that mandibular bone pattern, assessed on dental radiographs, could be used as a screening tool for osteoporosis and general fracture risk [23]. The mandibular premolar region has the fewest variations in anatomical size, shape, bone pattern and function, and is therefore the most suitable site for assessment of the bone trabecular pattern [24]. We hypothesized that the jawbone in obese individuals undergoing obesity treatment would reflect changes in their skeletal bone conditions. Hence, the aim was to investigate if changes in body mass index (BMI) results in changes of the mandibular trabecular bone structure.

## Materials and methods

A clinical study with a prospective, non-randomized, longitudinal design was registered on March 03, 2015, reg. no. NCT03152617. The study was carried out in Gothenburg, Region Västra Götaland in Sweden. The protocol was approved by The Regional Ethical Review Board of Gothenburg (reg. no. 673 − 14) and the Swedish Radiation Protection Committee (reg. no. 14–39). All procedures complied with the Helsinki Declaration and all participants signed an informed consent form.

### Participants

The present study comprised a subgroup of individuals from the BAriatric surgery SUbstitution and Nutrition study (BASUN), all referred for obesity treatment to the Regional Obesity Centre of Region Västra Götaland from primary health care. The baseline data collection was carried out from 3rd May 2015 to 19th December 2017 [25] before the obesity treatment, with scheduled follow-ups after 2, 5 and 10 years (main study *n* = 971). A broad interdisciplinary protocol was applied. The present subgroup of BASUN consisted of females aged 18–35 years at baseline (*n* = 117) [9]. The treatment (medical or surgical) was decided based on medical condition and patient’s preference. The follow-up of these women is intended to be finished before they enter the menopause. One hundred and thirteen women in the subgroup completed treatment and 66 of these women (58%) were re-examined at two years [26] with a mode delay of 2 months. The trabecular bone pattern was examined when the necessary radiographs were available (baseline *n* = 117, follow-up *n* = 66). The dental examinations were performed by a calibrated dentist and followed a standardized protocol [9]. All participants were fully dentated. A flow chart is provided as supporting material (Supplementary material S1).

### Intervention

All participants underwent an intervention for obesity, either medical or surgical. The medical treatment started with a period on a Very Low-Energy Diet (VLED) with subsequent food reintroduction and general advice [25,27] guided by licensed dieticians. The surgical treatment involved either Roux-en-Y gastric bypass (RYGB) or Sleeve Gastrectomy (SG) [28,29].

### Variables

The trabecular bone structure in the mandible was evaluated on intraoral digital periapical radiographs of the premolar area on the patient’s right side. None of the participants had periodontal problems that could interfere with the evaluations. Intraoral radiographs were used as they show more of the fine bone details than panoramic radiographs. The radiographs were exposed with a Focus DC X-ray machine with 0.4 s exposure time. Dürr Vista Scan Image plates plus were used. The digital images were stored in the Romexis^®^ system (Planmeca Oy, Helsinki, Finland) and saved as jpeg files for further analysis. The pictures were examined under dimmed lighting on an Olorin Vista Line^®^ monitor (Olorin AB, Kungsbacka, Sweden) by one of the authors (GJ) a dentist with extensive experience in the field. Previously, GJ and an oral and maxillofacial radiologist, performed a test–retest evaluation of mandibular trabecular bone using periapical radiographs with a very good result, intra-observer kappa = 0.97 and inter-observer kappa = 0.91 [30]. Another study gave similar results, intra-observer agreement 0.91 and inter-observer agreement 0.79 [31]. All assessments were performed blinded.

Two different methods were used to describe the trabecular bone structure as it appeared on the radiographs: Lindh’s index and Jaw-X.

The trabecular pattern of the alveolar bone was classified on the digital radiographs according to Lindh’s index as either ‘sparse trabeculation’ (large intertrabecular spaces, coded 1), ‘mixed sparse and dense trabeculation’ (small intertrabecular spaces cervically and larger spaces more apically, coded 2), or ‘dense trabeculation’ (small intertrabecular spaces, coded 3) [32, 33]. For the subanalyses, the trabeculation according to Lindh was dichotomized into dense (3 = 0) and mixed + sparse (1 + 2 = 1) (Supplementary material S2).

The Jaw-X software (Boneprox, Gothenburg, Sweden) created a binary filtered image from the jpeg file. The trabecular bone pattern in the region of interest was analyzed using a probe, fixed in size and shape but movable within the image (Supplementary material S3). The 20 largest intertrabecular spaces were identified and a Jaw-X value, which is the result of the size and intensities of the intertrabecular spaces, was computed. The value range is approximately between 3000 and 9000; values ≥ 6500 indicate a ‘risk of osteoporosis’, values between 6300 and 6500 indicate a ‘risk of osteopenia’, and values less than 6300 correspond to normal bone [34].

The main independent variable was BMI reduction from baseline to follow-up after two years (mean delay 2.5 months, mode value 2 months), expressed both as absolute figures and as percentages.

Additional variables were retrieved from the main BASUN study to describe the sample and for the control of confounders. The choice of these variables was grounded both on that they had significance for earlier similar analyses and on our own previous investigations (9, 26). The obesity treatment method, medical or surgical as described above, was registered. For the following variables, information was available both for baseline and follow-up: age in years, BMI as calculated from weight and height (kg/m^2^) and dichotomized BMI < 45 versus BMI ≥ 45 for the sub-analyses [9], pharmaceutical treatment (glucose-lowering treatment, blood pressure treatment, lipid-lowering treatment, treatment for anxiety/depression, treatment with antipsychotics, pain medication, hypothyroidism treatment, treatment for Attention Deficit and Hyperactivity Disorder), categorized as medication vs. no medication, physical activity (sedentary/some coded low activity vs. moderate/heavy coded high activity), ionized calcium in blood plasma (mmol/L), vitamin D in blood (nmol/L), and parathyroid hormone in blood (PTH, pmol/L). Smoking habits were self-reported (never smoked/stopped smoking ≥ 1 year ago vs. smoke occasionally/smoke daily). Socioeconomic status was likewise self-reported and represented by education (post-secondary schooling > 12 years vs. maximum secondary school ≤ 12 years) and cohabitation status (cohabiting vs. not cohabiting).

### Statistics

The data were checked and corrected for any input errors, and managed in Microsoft Excel^®^ (Microsoft Corporation, WA, USA). The statistical analysis was carried out using the SPSS^®^ software (Statistical Package for the Social Sciences), version 28 (IBM Corporation, NY, USA).

Descriptive statistics included frequencies, percentages, means, standard deviations (SD) and median values, when indicated. Chi-square, Student’s t-test and Kruskal-Wallis tests were used to analyze differences between categories. Bivariate Pearson correlations were used to explore associations between continuous variables. The impact of possible confounders on the difference in trabecular bone pattern between baseline and follow-up, as measured with Jaw-X, was explored using linear regression models. Crude analyses were followed by extended models including baseline information about age, socioeconomy (cohabitation status, educational level) and general health variables (medication, smoking, physical activity, ionized calcium, vitamin D, PTH) and the baseline value of the dependent variable (initial Jaw-X). The statistical significance level was set to 0.05.

## Results

Table 1 shows a description of the cohort by sociodemography and general health together with anthropometric measures and type of obesity treatment. At baseline, the mean BMI of the total group of 117 women was 42.2 (SD 4.0) and in the 66 women participating both times, BMI was 42.1 (SD 3.3) at baseline and at follow-up 33.1 (SD 7.5). However, the reduction at follow-up was only statistically significant in the surgically treated (mean BMI at follow-up 29.0, *p* < 0.001) and not in the medically treated patients (mean BMI at follow-up 39.4, *p* = 0.170) (not in tables). Among lifestyle variables, it was noted that a sedentary lifestyle was indicated by 40% of the participants before but merely by 17% after treatment.

**Table 1:** Sociodemographic, anthropometric and general health characteristics of participants at baseline and follow-up together with obesity treatment method

	Baseline *n* = 117	Baseline *n* = 66	Follow-up at two years *n* = 66
Age years^a^	27.9 (4.9)	28.1 (4.9) range18-35	30.6 (4.6) range 20–37
Height cm^a^	166.6 (6.2)	167.6 (5.9)	168.0 (0.1)
Weight kg^a^	117.5 (14.4)	118.7 (12.1)	93.0 (21.5)
BMI^a^	42.2 (4.0)	42.1 (3.3)	33.1 (7.5)
Cohabitation (no)^b^	52 (44.4)	31 (47.0)	24 (36.4)
Education ≤ 12 years^b^	72 (61.5)	38 (57.6)	35 (53.0)
Medication^b^	55 (47.0)	24 (36.4)	34 (51.5)
Smoking (yes)^b^	26 (22.2)	11 (16.7)	16 (24.2)
Physical activity^b^			
Sedentary	42 (38.9)	25 (39.7)	9 (17.0)
Some	58 (53.7)	35 (55.6)	33 (62.3)
Moderate	7 (6.5)	3 (4.8)	10 (18.9)
Heavy	1 (0.9)	-	1 (1.9)
Ionized calcium^a^ mmol/L	1.22 (0.03)	1.22 (0.03)	1.23 (0.04)
D-vitamine^a^ nmol/L	50.9 (20.6)	51.4 (21.7)	57.9 (19.2)
PTH^a^ pmol/L	4.2 (1.7)	4.4 (1.9)	3.95 (1.4)
Obesity treatment^b^			
Medical		26 (39.4)	
RYGB		26 (39.4)	
SG		14 (21.2)	

^a^ mean (SD) RYGB: Roux-en-Y Gastric Bypass PTH: parathyriodea hormone

^b^ n (%) SG: Sleeve Gastrectomy

nmol/L: nanomol per litre, pmol/L: picomol per litre

The obesity condition itself was related to bone density in the mandible. Thus, the group with dense trabeculation (Lindh’s index) had significantly higher BMI at baseline than the pooled group with mixed and sparse trabeculation (43.4 versus 41.4; *p* = 0.002) (Table 2a). The correlation between baseline BMI and Jaw-X was negative, as expected, but statistically non-significant (Pearson correlation − 0.099). However, the group with a BMI ≥ 45 had significantly lower mean Jaw-X values than the group with BMI < 45 (5657.8 versus 6015.3; *p* = 0.035) (Table 2b), indicating denser bone.

**Table 2:** a. Body Mass Index (BMI) related to bone trabeculation according to Lindh in obese women (baseline)

	*n*	BMI range	BMI mean (SD)	*p*
Mixed + sparse trabeculation	69	34.9–49.3	41.4 (2.9)	
Dense trabeculation	48	35.4–63.7	43.4 (5.1)	0.002

**Table 2:** b. Mandibular bone trabeculation as measured with Jaw-X related to categorized Body Mass Index: BMI ≥ 45 versus less in obese women (baseline)

	*n*	Jaw-X range	Jaw-X mean (SD)	*p*
BMI ≥ 45	21	4222–8158	5657.8 (938)	
BMI < 45	96	4505–8026	6015.3 (862)	0.035

The trabecular bone pattern of the mandible for individuals participating both at baseline and at follow-up (*n* = 66), is shown in Table 3. At baseline, dense trabecular bone classified according to Lindh was found in 43.9% of the women, while 12.1% (*n* = 8) had sparse trabeculation. At follow-up, only one individual was classified as having sparse trabeculation while the majority was classified as having mixed trabeculation (56.1%, *p* < 0.001). The number in the dense bone group was basically unchanged with only one individual less at follow-up. There was no difference between medically and surgically treated patients (*p* = 0.381).

The mean Jaw-X value in the total group at baseline was 5935.2, which decreased to a mean of 5627.7 at follow-up (*p* = 0.009). According to the Jaw-X values, 69.7% had normal bone density, 9.1% a risk of osteopenia and 21.2% a risk of osteoporosis. At follow-up, the group with normal Jaw-X values < 6300 had become larger (80.3%). However, within the two treatment groups, no significant differences could be identified from baseline to follow-up (medical treatment *p* = 0.055, surgical treatment *p* = 0.082, not in table). No difference could be noted between medically and surgically treated patients (*p* = 0.902, not in table).

**Table 3:** Trabecular bone structure of the mandible in participants at baseline and at follow-up two years after obesity treatment

	Baseline *n* = 66	Follow-up at two years *n* = 66	*p*
Bone trabeculation according to Lindh^a^			< 0.001
Sparse^a^	8 (12.1)	1 (1.5)	
Mixed^a^	29 (43.9)	37 (56.1)	
Dense^a^	29 (43.9)	28 (42.4)	
Jaw-X^b^	5935.2 (838.5) range 4505–8026	5627.7 (666.4) range 4372–7050	0.009
Jaw X: ≥6500^a^	14 (21.2)	7 (10.6)	
Jaw X: ≥6300 - <6500^a^	6 (9.1)	6 (9.1)	
Jaw X: <6300^a^	46 (69.7)	53 (80.3)	

^a^ n (%)

^b^ mean (SD)

In Table 4, the Jaw-X mean values are related to the three trabeculation groups categorized according to Lindh. The Kruskal-Wallis test showed that there were significant differences, both at baseline and at follow-up, between the mean Jaw-X values in the three trabeculation categories on both occasions *p* < 0.001.

**Table 4:** Jaw-X mean values in the three trabeculation groups categorized according to Lindh at baseline and at two years after obesity treatment

		Baseline Jaw-X mean value	Follow-up at two years Jaw-X mean value
Bone trabeculation according to Lindh			
Sparse		7482.8	5864.0
Mixed		6021.2	5695.6
Dense		5422.2	5494.6
*p* at each point in time		< 0.001	< 0.001

Table 5 presents the impact of possible confounders on the relationship between the percentage of BMI reduction from baseline to follow-up (predictor) and the difference in Jaw-X (outcome/response). Baseline information about the covariates was used, as this was the starting point and assumed to have prevailed during most of the period. All models, except the crude model, were statistically significant. The β value increased in the extended models when the type of obesity treatment (medical or surgical) and socioeconomic status were added as covariates. Notably, the type of treatment was not statistically significant per se. Of the other covariates, age and the initial Jaw-X value were statistically significant in all models, as was the level of ionized calcium included in the final model. No other covariates were statistically significant in any model. Parallel identical analyses with absolute numbers for BMI reduction as the predictor showed the same pattern.

**Table 5:** Difference in trabecular bone structure as measured with Jaw-X from baseline to follow-up dependent of percentage of BMI reduction. Linear regression models.

Response	Adjustment	Coefficient β	p for coefficient	95% CI for coefficent	*R* for model	*R* ^2^ for model	Adjusted *R*^2^ for model	F for model	*p* for model
Differential in Jaw-X	Crude	5.55	0.396	-7.44–18.54	0.106	0.011	-0.004	0.729	0.396
	Age (baseline)	6.08	0.314	-5.89–18.04	0.418	0.175	0.149	6.67	**0.002**
	Age, baseline Jaw-X	4.83	0.248	-3.45–13.11	0.782	0.612	0.593	32.58	**< 0.001**
	Age, baseline Jaw-X, obesity treatment	13.91	**0.042**	0.53–27.29	0.794	0.630	0.605	25.93	**< 0.001**
	Age, baseline Jaw-X, obesity treatment, SES	14.64	**0.035**	1.08–28.20	0.798	0.637	0.600	17.25	**< 0.001**
	Age, baseline Jaw-X, obesity treatment, SES, general health	9.81	0.173	-4.47–24.09	0.808	0.652	0.565	7.50	**< 0.001**

Obesity treatment: medical or surgical

SES = socioeconomic status: cohabitation, education

General health: medication, smoking, physical activity, ionized calcium, D-vitamin, parathyroid hormone

## Discussion

A main finding of the study was that a high percentage of obese young women had dense trabeculation in the jaws before treatment. This group with dense trabeculation had significantly higher BMI than the pooled group of patients with mixed and sparse trabeculation, measured by Lindh’s index. Also, the method using Jaw-X showed that a high BMI (≥ 45) was associated with denser bone compared with a lower BMI. At the examination two years after the treatment, fewer were classified with sparse bone. The association between BMI reduction and the difference in bone structure over the period was influenced by the covariates applied. The method of treatment—medical or surgical—was not found to be significant for the outcome.

### Strengths and limitations of the study

The prospective design can be considered a strength of the study. The participation rate in longitudinal studies is always a concern and only three out of five of the individuals in the baseline study participated in the follow-up after two years. The potential loss of power may have influenced the results and more statistically significant associations might have been achieved if a larger number had participated. A randomized design would have been desirable but both the patients’ medical condition and wishes were considered, primarily for therapeutic but also for ethical reasons. Also, it must be noted that only young women were recruited to the odontological BASUN sub-study, as the follow-ups are planned to be completed before the menopause with its consequent hormonal changes [35], and because most of the individuals seeking obesity treatment are women [36].

We could not demonstrate any difference in bone trabeculation between medical and surgical treatment groups over the observation period, which might have been expected. Possibly the time was too short or the groups should have been larger.

Mandibular bone density has been used as a proxy for systemic bone density including the hip and spine [34], and we used dental radiographs for the same reason. The classification for bone trabeculation according to Lindh et al. (1996) [32], has been validated with several objective methods for the prediction of osteoporosis [37, 38], and for future fracture risk [39]. The Jaw-X digitized method predicted fracture in the study of Jonasson & Billhult [34], but not in a study of Hassani-Nejad et al. [30]. In another study, Jaw-X showed sparser trabeculation in patients with Chron’s disease compared with a matched control group [40]. An overview of mandibular bone trabeculation in earlier studies of young women is shown in Supplementary material S4 [30, 31, 34, 39, 40]. None of these studies were performed on an obese population and had a smaller proportion (4-25%) with dense trabeculation than in our study (Lindh’s index, 48 of 117; 41%). Comparisons between analogue and digitized methods for measuring bone density have been made with varying results [31, 37, 41]. However, in the current study, the two methods gave similar results and complemented each other.

The WHO criterion for obesity is BMI equaling 30 or higher. However, recent reports show that more extreme BMI values are increasing in number [1]. In two earlier publications by our group from the same cohort, we noted that oral health problems, such as increase in dental caries, appeared only at considerably higher BMI values [9, 26]. This was the reason for the choice of cutoff at BMI = 45 for the analysis of bone trabeculation in the current study, which also turned out to have significance regarding the relationship between BMI and the Jaw-X value. A possible factor to consider is the amount of soft tissue in the area in question, which has been shown to be important for other bone measurement methods, e.g., Dual energy X-ray Absorptiometry, DXA [42]. After successful obesity treatment, the adipose tissue also decreases in the jaw area. However, the amount of soft tissue is of no importance when analyzing bone structure, as in our study [38]. Using bone structure instead of bone mass and thereby avoiding the influence of soft tissue can be considered a strength of our study.

No statistically significant difference in Jaw-X over time between medically and surgically treated patients was found in the study, which might have been expected. A possible explanation for this may be the small weight change in the medically treated group.

### Interpretation

The association between obesity level and bone trabeculation that was shown in the study is in line with previous research. A systematic review revealed that high levels of obesity generally increased bone mineral density (BMD) [43]. Likewise, dense trabeculation in the mandibular bone has previously been shown to be a strong indicator of high BMD in the skeleton, whereas sparse trabeculation is combined with low BMD [44]. Heavy loading of the skeleton of various causes has been shown to result in greater bone mass [45, 46] and effects of loading are seen in the same way in the jaws [47]. These mechanisms could possibly explain our finding of denser bone at higher BMI values.

The results at follow-up indicated a trend towards improvement of the bone structure; however, without statistical significance. In contrary, a Brazilian study showed visually impaired bone pattern in the mandible six months after bariatric surgery but the individuals had a higher BMI and the measurement methods and analyses differed from our study [48]. Also, our findings are in contrast to studies on other bones in the body with Dual energy X-ray Absorptiometry (DXA) showing decreasing bone density after bariatric surgery [49, 50]. However, Elias et al. (2014) found that total BMD did not change in the first year after a Roux-en-Y gastric bypass, but skull BMD was significantly reduced compared with baseline [49], and a review found that fracture risk is dependent on the time elapsed since surgery and increases after about 3 years [50]. A possible reason may be that both general and local factors are likely to be important for the bone structure in the jaw [38]. The overall lifestyle, including physical activity should be considered. Weight loss facilitates physical activity, and our group of young women was encouraged to exercise to a greater degree. This may have had a positive effect on the jawbone structure, which could explain why the mandible did not show any deterioration in appearance. However, it should be noted that the measure of physical activity was self-reported, entailing a risk of so-called social desirability, which has been shown especially for Roux-en-Y gastric bypass patients [51]. All participants received diet counseling in the study [25] and all were fully dentated, enabling effective chewing with positive loading of the jawbone [52]. It might be speculated that the participants chewed their food more carefully after treatment, which could possibly lead to a denser jawbone structure. Also, decreases in DXA-measured BMD when body weight and/or fat mass decrease, as demonstrated in previous clinical studies, might be due to systematic errors in the DXA method [53]. The potential biological mechanisms driving the relationships between obesity, its treatment and bone structure should be considered overall. Menopause, implicating oestrogen deficit, and increasing age are probably such mechanisms. Possibly there is a threshold to cross before negative systemic factors adversely affect bone trabeculation. It is also possible that the follow-up time was too short to discern long-term trends, although bone replacement may occur quite quickly [19, 54]. Hence, the findings suggest that longer follow-up periods are needed.

A general increased fracture risk after surgical treatment has been reported [13–16] and previous research has indicated that dental radiographs can be used for screening of this risk [23]. Therefore, if BMI reductions are closely followed by changes in mandibular bone structure, dentists can use sparse mandibular trabeculation as an indicator for increased skeletal fracture risk (hip, forearms, pelvis, but not the mandible itself, where fractures in principle only occur after trauma). A collaboration between the dental and medical professions could then be established for identifying women before the first fracture occurs.

The relationship between weight loss and bone structure measured by Jaw-X showed an interesting picture when examined more closely in the regression models. As mentioned above, the crude association between them showed no significant relationship but was affected by the possible confounders that were included so that the models became significant. The most important factors were age and bone structure (Jaw-X value) at baseline. This seems logical and is in line with other studies in the field [55, 56]. As a whole, it is interesting that most of the covariates applied were not statistically significant per se. Their selection was based on previous studies [9, 26], but there is a need to go further and investigate other factors, such as different hormones in the blood.

The causal relationships between BMI reduction achieved through obesity treatment and bone trabeculation in the lower jaw may be discussed, in part, on the basis of the laboratory measurements used as covariates in the regression models. For example, levels of vitamin D in the blood increased after treatment, which was also observed in the main BASUN cohort with no difference between medical and surgical treatment [11]. This might be explained by the fact that there is less fat in the tissue to harbor the vitamin after treatment [57]. Higher BMI is also more likely to be associated with lower calcium levels for the same reason. However, the levels of ionized calcium remained unchanged during our study period, which is also consistent with the main study [58]. Calcium was the only covariate that was consistently statistically significant on its own when included in the regression models, indicating its importance for the analyses.

To sum up, using mandibular bone structure as a proxy for skeletal BMD, no deterioration was seen the first years. However, it is important for women that is treated for obesity to continue with new good habits like physical activity and healthy nutrition, to avoid malnutrition and increased fracture risk. Overall, the mechanisms behind the effect of BMI reduction in the jawbone remain to be explored.

## Conclusion

Before obesity treatment, high BMI was associated with dense bone trabeculation in the jaw. The group with sparse bone had decreased at follow-up. The association between BMI reduction and bone trabeculation was influenced by individual and medical factors.

## Electronic supplementary material

Below is the link to the electronic supplementary material.

Supplementary Material 1

Supplementary Material 2

Supplementary Material 3

Supplementary Material 4

## Data Availability

The data that support the findings of this study are available from the corresponding author upon reasonable request.

## Declarations

### Clinical trial

The trial was prospectively registered on March 03, 2015; NCT03152617.
