# Supplementary material for: Mandibular trabecular bone pattern before and two years after medical or surgical obesity treatment in young Swedish women
Source: Clin Oral Investig. 2025 Jan 12;29(1):57. doi: 10.1007/s00784-024-06142-y (PMC11725539; doi:10.1007/s00784-024-06142-y)
Supplement: Supplementary file 1 — Supplementary Material 1 [file 784_2024_6142_MOESM1_ESM.pdf]

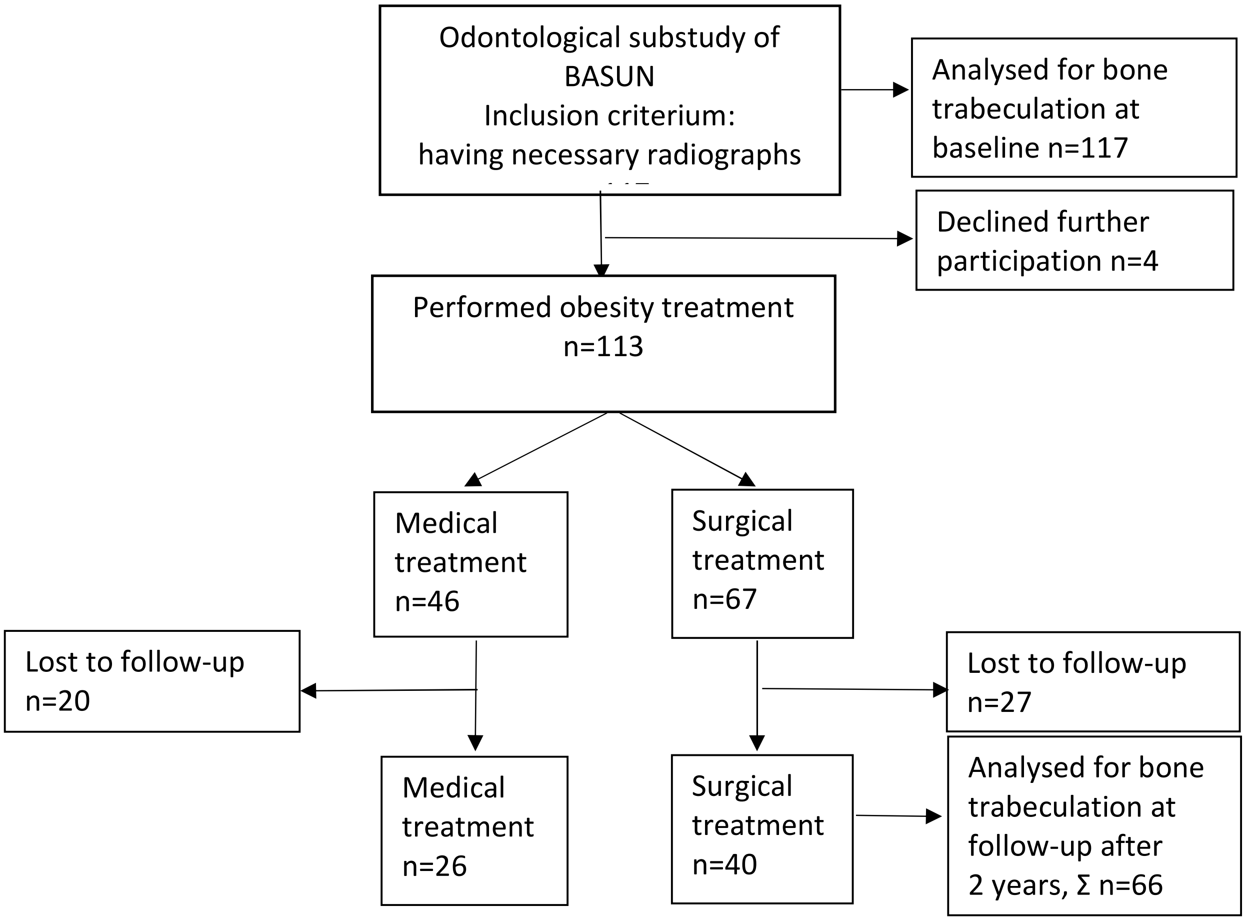

BASUN: BAriatric surgery SUbstitution and Nutrition study
Modified from a figure earlier published:
Taghat N, Mossberg K, Lingström P, Petzold M, Östberg AL. Impact of Medical and Surgical Obesity Treatment on Dental Caries: A 2-Year Prospective Cohort Study. Caries Res. 2023;57(3):231-242. doi: 10.1159/000533609. Epub 2023 Aug 16. PMID: 37586350.

Figure S1.
Article title: Mandibular trabecular bone pattern before and two years after medical or surgical obesity treatment in young Swedish women
Authors: Östberg AL, Wallenius V, Taghat N, Jonasson G.
Affiliation and e-mail of the corresponding author: Department of Behavioural and Community Dentistry, Institute of Odontology, Sahlgrenska Academy, University of Gothenburg, Gothenburg, Sweden; anna-lena.ostberg@odontologi.gu.se
