# Supplementary figures and images for: Mandibular trabecular bone pattern before and two years after medical or surgical obesity treatment in young Swedish women
Source: Clin Oral Investig. 2025 Jan 12;29(1):57. doi: 10.1007/s00784-024-06142-y (PMC11725539; doi:10.1007/s00784-024-06142-y)

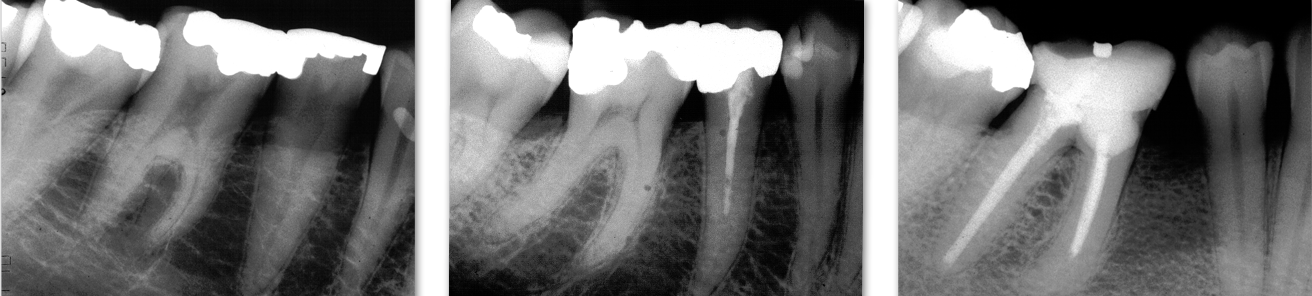

Supplement: Supplementary file 2 — Supplementary Material 2 [file 784_2024_6142_MOESM2_ESM.tif]

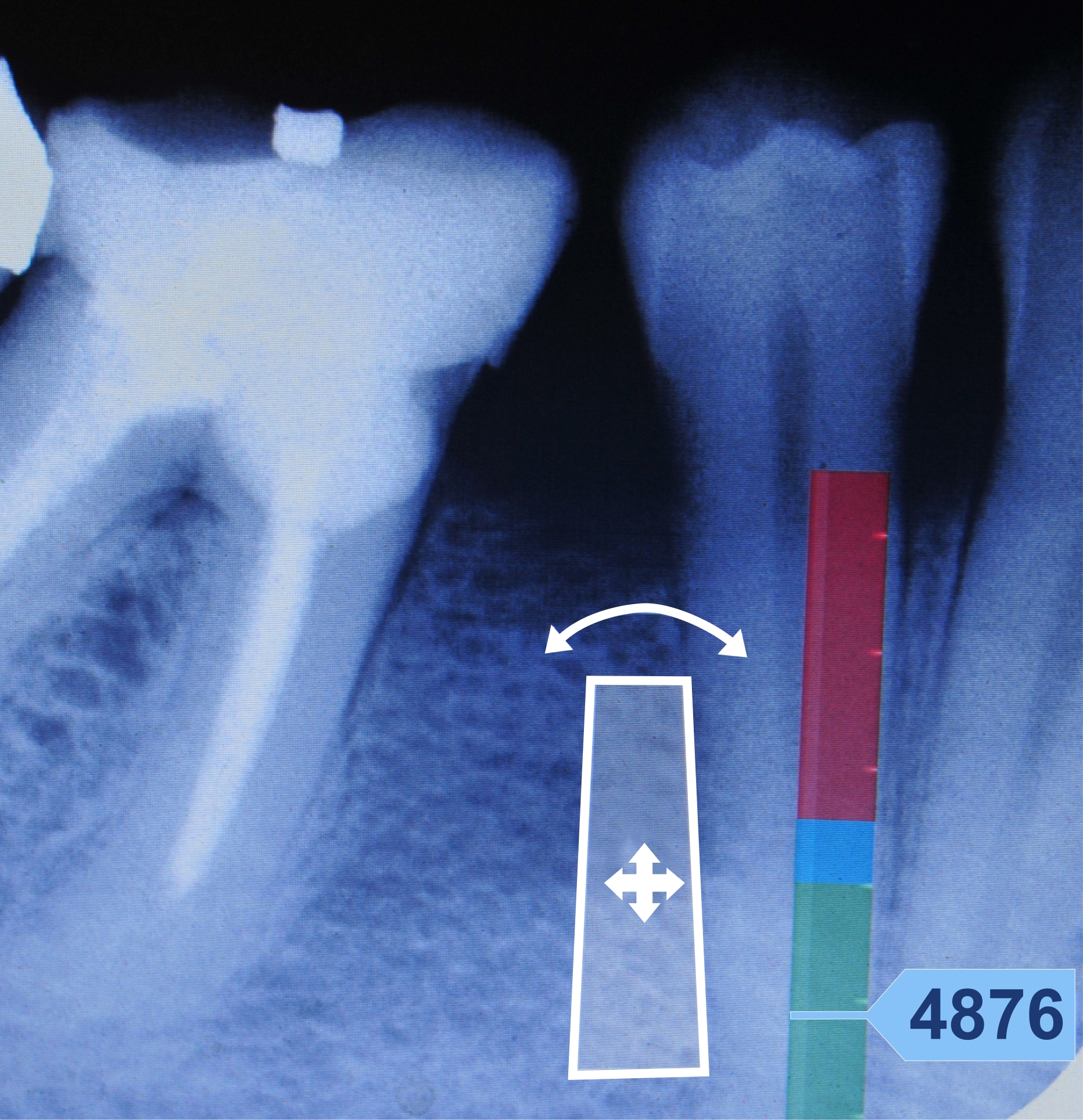

Supplement: Supplementary file 3 — Supplementary Material 3 [file 784_2024_6142_MOESM3_ESM.tif]
