# Supplementary material for: Mandibular trabecular bone pattern before and two years after medical or surgical obesity treatment in young Swedish women
Source: Clin Oral Investig. 2025 Jan 12;29(1):57. doi: 10.1007/s00784-024-06142-y (PMC11725539; doi:10.1007/s00784-024-06142-y)
Supplement: Supplementary file 4 — Supplementary Material 4 [file 784_2024_6142_MOESM4_ESM.docx]

Supplementary material S4. Studies of mandibular bone trabeculation in young women.

| Authors | Reference | Sample characteristics | *No*, age | Jaw-X mean (SD), range | Dense^a^% | Mixed^a^% | Sparse^a^% |
| --- | --- | --- | --- | --- | --- | --- | --- |
| Jonasson & Billhult 2013 | 32 | Females, dental patients | *136*, 35-94 | 6663.1 (899.4), 4128-8625 | 11.9 | 73.1 | 15 |
| Hassani-Nejad et al., 2013^b^ | 28 | Females, general population | *150,* 80 | 6132 (627), 4500–7772 | 30 | 46.7 | 23.3 |
| Jonasson et al., 2014 | 33 | Both genders  Patients with Chron’s disease  Controls, matched sex and age | *49,* 23-61  *49,* 23-61 | 7086 (954), 5580–9048  5117 (947) ,2988–7448 | 24.4  18.4 | 44.4  69.4 | 31.1  12.2 |
| Sundh et al., 2017 | 34 | Females, general population  Baseline  Follow-up after 12 years | *499,* 50-66  *412*, 62-78 | ̶̶  ̶ | 17  13.4 | 45.5  38.4 | 37.5  48.3 |
| Elleby et al., 2021^c^ | 29 | Females, general population | *237,* 18-36 | 5348.4 (864.4), 2774-7702 | 3.8 | 94.1 | 2.1 |

^a^ Lindh’s index

^b^ Female sample extracted by one of the authors

^c^ Young female sample extracted by one of the authors
